# Risk factors of acute malnutrition among children aged 6–59 months enrolled in a community-based programme in Kurigram, Bangladesh: a mixed-method matched case-control study

**DOI:** 10.1186/s41043-019-0192-2

**Published:** 2019-11-27

**Authors:** Monsurul Hoq, Masum Ali, Ashraful Islam, Charulatha Banerjee

**Affiliations:** 1 Terre des hommes, Dhaka, Bangladesh; 2 Murdoch Children’s Research Institute, Melbourne, Australia; 3 Hellen Keller International, Dhaka, Bangladesh; 4 ENN, Kolkata, West Bangal, India

**Keywords:** Risk factors, Child malnutrition

## Abstract

**Background:**

Risk factors of acute malnutrition in Bangladesh are well documented. However, due to regional variations in prevalence of acute malnutrition, it is important to explore the risk factors specific to the region, for designing and implementing public health interventions.

**Methods:**

A mixed-method matched case-control study was conducted in the Kurigram district of Bangladesh. Community perspectives on causes of acute malnutrition were collected from 75 purposively selected caregivers through interviews, focus group discussions and informal group discussions. The data was analysed manually by coding and sub-coding according to different themes. Caregivers of 52 malnourished and 95 well-nourished children matched in age group and sex with the malnourished children, were interviewed using a structured questionnaire. The conditional logistic regression analysis was performed to identify the risk factors of acute malnutrition.

**Results:**

Caregivers perceived inappropriate feeding practice as a major cause of acute malnutrition whereas birth order (first child OR 0.3, 95% CI 0.09, 0.96), number of family members (OR 1.30, 95% CI 1.02, 1.65), illness in the last 2 weeks (OR 3.08, 95% CI 1.13, 8.42) and access to hygienic latrine (OR 0.25, 95% CI 0.07, 0.82) were also associated with acute malnutrition among children under five in Kurigram.

**Conclusions:**

Community awareness on infant feeding practices and family planning, management of childhood illness and access to hygienic latrine facilities should be prioritised to prevent acute malnutrition in the northern districts.

## Background

Childhood acute malnutrition, in the form of wasting defined by weight-for-height *z*-scores (WHZ) or mid upper arm circumference (MUAC), is a major public health concern in developing countries. According to World Health Organization (WHO) nearly 20 million children are suffering from acute malnutrition and more than 70% of the world’s children with wasting live in Asia, most in south-central Asia [1]. Levels of malnutrition are also of concern in Bangladesh as 16% of children aged under five from rural areas suffer from wasting defined by WHZ < − 2SD [2]. The prevalence of wasting varies between different regions of Bangladesh, and riverine islands account for the most number of cases [3].

The causal path of acute malnutrition is very complex, whereby biological, cultural and socio-economic factors are interrelated. As described by the UNICEF casual framework, the causes of malnutrition are divided into immediate, underlying and basic causes; inadequate dietary intake and disease are the immediate causes; household food insecurity, inadequate care and poor sanitation and hygiene practice are underlying causes; other socio-economic characteristics are classed as basic causes of malnutrition in developing countries [4]. Nutritional causal analysis conducted by Action Contre La Faim (ACF), in Sathkira (Southern Bangladesh), identified poor infant and young child feeding (IYCF) practice, low levels of psychosocial care for women, poor health seeking behaviour, low-income opportunities and poor water sanitation and hygiene (WASH) practices as risk factors [5]. A secondary data analysis of the Bangladesh Demographic and Health Survey (BDHS) data by Rygan and Khan indicated that interval from the previous birth, birth size, mother’s body mass index at the time of birth, and parents’ education were the main contributing factors of under-five malnutrition [6]. A study of young children admitted to a diarrhoea treatment facility in Bangladesh also revealed that parental education, economic characteristics, child-feeding practices and birth order were important risk factors for severe underweight [7].

Although the main factors contributing to malnutrition in Bangladesh are well documented, the factors varies by region and over time. The purpose of this study is to identify risk factors of acute malnutrition in the Kurigram District of northern Bangladesh and assist policy makers in designing and targeting preventive intervention projects addressing the main factors specific to this area.

## Methods

A mixed-method study was conducted from July 2013 to June 2014 in three Unions of Kurigram District, situated in the northern region of Bangladesh. Initially, 18 caregivers of children aged under five years were interviewed, six focus group discussions (FGDs) with a total of 42 women, and an informal group discussion (iFGDs) with 15 people residing in the study area were conducted to explore community perception of the causes of acute malnutrition. All participants of the qualitative study were selected purposively (i.e. not randomly) from the community awareness session of Terre des hommes (Tdh), who had received health messages on exclusive and continued breast feeding, appropriate complementary feeding, correct handwashing practice, safe drinking water, hygienic latrines, delaying age at marriage, and family planning methods, or from the outdoor waiting area of Tdh primary clinic in Kurigram but were not beneficiaries of the Tdh community health programme, based on their availability, interest to participate in the study and was a caregiver of under-five year old child [8]. The notes from interviews, FGD and iFGDs were initially analysed separately by coding and sub-coding according to different themes and later summarised after triangulation.

Following the qualitative study, a matched case-control study was conducted to identify the risk factors of acute malnutrition among children aged 6–59 months. Cases were defined as children with WHZ < − 2SD and/or MUAC < 125 mm, and controls were children with WHZ ≥ − 2SD and MUAC ≥ 125 mm who were residing in the same neighbourhood as the cases. The cases and controls were matched in two age groups, i.e. either 6 to 23 months or 24 to 59 months, and by sex. All the children (*n* = 52) enrolled in the community management of acute malnutrition (CMAM) project of Tdh during February 2014 to May 2014 were selected as cases, and 95 children from the same neighbourhood of the cases were selected as controls with a ratio of 1: 2 between case and control. Initially controls were listed based on the information provided by the caregivers of malnourished children, and selected after household visits. If more than two controls were identified, controls with minimum age difference with case were selected. However, for nine cases only one control was identified. All caregivers selected, participated in the study.

Data was collected from the child’s primary caregiver using a structured questionnaire. Based on the UNICEF conceptual framework and literature review of causes of malnutrition, data was collected on the following potential risk factors; socio-economic and demographic factors: parental education, father’s occupation, mother’s occupation, mother’s body mass index, family income, mother’s age at marriage, number of living children, birth order, number of household members, total land owned, household food security; dietary factors: infant and young child feeding practices, household food taboos; environmental factors: household drinking water source, water purification system, type of toilet facility, health care seeking behaviour; and immunization factors: measles and vitamin A coverage.

The in-depth interviews and FGDs were conducted by a trained Research Assistant, and structured questionnaires were administered by Monitoring Assistants of Tdh. Mothers’ and children’s weight was measured using electronic scales (Digital Lithium Scale, HD-318, Highest 150 kg, China) with precision of 100 g. Height or length was measured using locally manufactured height measurement boards to the nearest mm by Monitoring Assistants. All measurements were made in line with the WHO standard protocols [9]. All growth indexes were calculated using WHO growth standard of 2006 using ENA software [10]. Research Assistants and Monitoring Assistants involved in the study received training on data collection tools, were supervised during data collection and validated by checking records and re-visiting 5% of the sample.

Some variables were categorised for more specification. Age of the child was categorised into two groups, i.e. 6–23 months and 24–60 months considering the higher likelihood of younger children becoming malnourished. Birth order of the child was categorised as first child and not first child since culturally preference is given to the first child. Parents’ education was categorised as illiterate and literate, where a person unable to read and write was considered illiterate. In order to explore taboos related to feeding practice, a series of questions were asked to caregivers of both cases and controls based on the findings of qualitative study. The responses were then analysed into two categories as family who believed in food taboos and who did not. The household water source was classified as improved only if it was a tube-well with platform. Similarly, sanitary latrine with water-sealed pan was classified as a hygienic latrine. Household food security was measured by household food insecurity access scale (HFIAS) ), household hunger scale (HHS) and food consumption score (FCS) [11–13]. Households were later categorised as food insecure if found with any level of insecurity. Infant and young child feeding practices were measured according to the indicators recommended by the WHO [14].

The quantitative data was analysed using STATA 12 [15]. Bivariate and multivariate conditional logistics regression analysis was conducted to compare different characteristics between malnourished and well-nourished children taking the age group and sex matching into consideration [16]. The socio-demographic, food security, health and hygiene characteristics, for which there were enough evidence of an association with acute malnutrition based on the bivariate analysis, were considered for multivariate analysis.

## Results

The proportion of girls, and children aged less than 24 months was similar in both case and control groups. The children with acute malnutrition had lower mean weight-for-age *z*-score than children without acute malnutrition. However, there was not enough evidence of a difference in mean height-for-age z-score (Table 1). Among the cases 15.4% (*n* = 8) was the first child compared with 41.0% (*n* = 39) among controls (Table 1).

**Table 1:** Socio-demographic characteristics by nutritional status of the study children and measures of association, Kurigram, Bangladesh, Feb–May 2014

Characteristics	Children with acute malnutrition^a^ (*n* = 52)	Children without acute malnutrition (*n* = 95)	Odds ratio* (95% CI, *p* value )
Sex of the child			
Female, *n* (%)	25 (48.1 %)	48 (50.5 %)	0.92 (0.50,1.71; 0.793)
Age of the child			
< 24 months, *n* (%)	34 (65.4 %)	64 (67.4 %)	1.69 (0.13, 21.13; 0.685)
Weight-for-age *z*-score (mean ± SD)	− 2.9 ± 1.0	− 1.5 ± 0.9	0.12 (0.04, 0.30; < 0.001)
Height-for-age *z*-score (mean ± SD)	− 1.7 ± 1.8	− 1.6 ± 1.3	0.95 (0.75, 1.20; 0.665)
Birth order			
First child, *n* (%)	8 (15.4 %)	39 (41.0 %)	0.24 (0.10, 0.60; 0.002)
Mother’s age at marriage (in years, mean ± SD)	14.9 ± 2.2	15.0 ± 2.3	0.98 (0.83, 1.16; 0.835)
Mother’s age (in years, mean ± SD)	26.6 ± 7.1	23.9 ± 5.2	1.09 (1.02,1.16;0.010)
Mother’s BMI (mean ± SD)	19.1 ± 3.0	19.9 ± 2.7	0.90 (0.79, 1.02; 0.094)
Mother’s education			
Illiterate, *n* (%)	17 (32.7 %)	25 (26.3 %)	1.34 (0.67, 2.70; 0.401)
Father’s current age (in years, mean ± SD)	34.4 ± 8.9	30.8 ± 6.2	1.08 (1.02, 1.14; 0.004)
Father’s education			
Illiterate, *n* (%)	27 (51.9%)	38 (40.0 %)	1.64 (0.79, 3.43; 0.182)
Family income (Taka, mean ± SD)	5888.5 ± 2468.6	6257.9 ± 2681.6	1.00 (1.00, 1.00; 0.477)
Total family member (mean ± SD)	6.1 ± 2.4	5.2 ± 2.0	1.27 (1.06, 1.53; 0.011)

^a^Acute malnutrition was defined by weight-for-height *z*-score < − 2SD and/or mid upper arm circumference < 125 mm

*p value and odds ratio were estimated using conditional logistic regression taking age group and sex matching into consideration

In both case and control groups, the mean age of mothers at marriage was 15 years, and one in every three mothers was illiterate (Table 1). However, there was not enough evidence that mothers’ age at marriage or literacy status was associated with nutritional status of the child (Table 1). All caregivers mentioned having wanted to continue studying in school but the social norms dictated that families of girls who were educated and were older would need to pay more gifts in terms of property or money to her husband at the time of marriage, a practice prevalent in many communities in South Asia including Bangladesh, and referred to as dowry. Illiteracy and low family income also play a role in this as parents did not want to waste precious resources on a girl’s education. As stated by a mother *— “My daughter will leave my house someday, she will not support me in the future so it is good to marry her off early and there is no need to spend on her education*”.

The importance of exclusive breast feeding and continued breast feeding along with timely introduction of family foods was widely shared by the caregivers. One of the breast-feeding mother mentioned that, “*I did not fed my first three children within the first 24 hours but I tried to breast fed the last child immediately and continued thereafter*”. A few caregivers of malnourished children reported insufficient or complete lack of breast milk. Descriptive analysis of breast feeding practice of children aged 6–23 months enrolled as case or control revealed that among 107 children from the same number of women, only 2 (2%) were never breastfed, 91 (87%) were immediately put to breast after birth, and 84 (93%) were continuing breastfeeding.

The caregivers informed that most children were served meals two to three times only, along with other members of the family. Among the 107 children aged 6–23 months enrolled in the quantitative study, only 46 (43%) had minimum adequate meal frequency and only 9 (8%) had a minimum acceptable diet in line with WHO’s recommended infant and young child feeding practice. As a possible reason the findings of qualitative study indicated that caregivers were unfamiliar with the concept of a diversified diet. Moreover, knowledge of nutritious food varied greatly among this group. Caregivers informed that they prepared meals using available resources and due to a lack of money they were unable to buy meat and other food items from the market. Most of the caregivers shared their concern about child’s lack of appetite. It was observed that in most cases children were served the same food at breakfast and lunch. The poor infant and young child feeding practice could also be linked to food insecurity as children with acute malnutrition were more likely from food insecure households compared with children without acute malnutrition (OR 2.57, 95% CI 1.09, 6.07; *p* = 0.31) based on the HFAIS scale (Table 2). However, no association was found based on HHS or FCS scale (Table 2).

**Table 2:** Food taboo, food security, immunization, illness and care seeking, access to water and sanitation of household by nutritional status of the study child and measures of association, Kurigram, Bangladesh, Feb–May 2014

Characteristics	Children with acute malnutrition^a^ 52, *n* (%)	Children without acute malnutrition 95, *n* (%)	Odds ratio* (95% CI, *p* value )
Food taboo			
Yes	33 (63.5%)	72 (75.8 %)	2.02 (0.88, 4.65; 0.097)
Household hunger scale			
Moderate or serve hunger	39 (75.0 %)	78 (82.1 %)	1.60 (0.64, 4.01; 0.312)
Food consumption score			
Food secure	27 (51.9 %)	52 (54.7 %)	1.11 (0.56, 2.20; 0.770)
Household food insecurity access scale			
Food secure	10 (19.6 %)	34 (36.2 %)	2.57 (1.09, 6.07; 0.031)
Received vitamin A			
Yes	45 (86.2 %)	84 (88.4 %)	0.83 (0.25, 2.73; 0.761)
Illness in the last 2 weeks			
Yes	40 (76.9 %	52 (54.7 %)	2.98 (1.30, 6.78; 0.009)
Health service provider			
Formal	10 (25.0%)	7 (14.3 %)	1.58 (0.42, 5.99; 0.500)
Household water source			
Tube-well with platform	12 (23.1 %)	23 (24.2 %)	0.97 (0.41, 2.29; 0.942)
Purify drinking water			
Yes	3 (5.8 %)	2 (2.1 %)	0.21 (0.02, 2.17; 0.193)
Household toilet facility			
Hygienic latrine	10 (19.2%)	32 (33.7 %)	0.34 (0.12, 0.95; 0.039)

^a^Acute malnutrition is defined by weight-for-height *z*-score < − 2SD and/or mid upper arm circumference < 125 mm

*p value and odds ratio were estimated using conditional logistic regression taking age group and sex matching into consideration

Food taboos were reported by the caregivers at every stage of pregnancy and after child birth. During the antenatal period, food was restricted. It was believed that if the woman gained weight, the size of the new born would be big increasing the risk of difficulties at birth. During the post-natal period, women were confined to a single room for 40 days after child birth and easily available nutritious foods such as duck’s egg, fishes and pulses were not fed. Some women also reported not having warm food; the perception being that hot rice would affect the child. Food was also restricted to women based on the condition of the child. If a child was ill, the caregiver was not fed fish and potatoes, both of which were easily available sources of protein and carbohydrate in Kurigram and also form a big part of an everyday family meal. It was also assumed that restricting the food of the mother would make the child well. The quantitative study revealed that 63.5% of case families compared with 75.8% of control families reported food taboos (OR = 2.02, 95%CI 0.88, 4.65; *p* = 0.097). However, there was not enough evidence of difference in proportion of caregivers who believe in food taboos between case and control group (Table 2).

Most of the children with acute malnutrition were suffering from illness in the last 2 weeks compared with children without acute malnutrition, and the association was statistically significant (OR 2.98, 95% CI 1.30, 6.78; *p* = 0.009). Fever (63%) and diarrhoea (16%) were the most frequent illnesses reported. The findings were similar to the perception of the caregivers as illness was attributed as a cause of the child’s poor nutritional condition. According to the caregivers, respiratory illnesses such as cough, pneumonia, asthma and skin diseases were common in winter, while dysentery and chicken pox were common in summer. Fever and diarrhoea were reported in both seasons. Newborn illnesses were attributed to the effect of an “evil eye”, i.e. cursed by a malevolent glare, and the remedy was a priest or a traditional healer. Quoting a mother “*My child was not able to feed at my breast so everyone said that a spirit has attacked him. So I consulted a priest who gave me an amulet for 7 days which made no difference*”.

In spite of the knowledge that illness causes malnutrition, proper care seeking for illness was very poor. Caregivers most frequently depend upon advice from elders in their family and community. The traditional healer came next followed by some over the counter medication from the pharmacy. A few went to a primary healthcare centre or a hospital (Table 2). Most caregivers were unaware about the primary healthcare centres. Of the few that sought care from the primary health centre, some complained about the distance they had to travel, and lack of medicine at the facility.

Caregivers of well-nourished children recognised that an unclean environment in the home and poor hand washing practices were causes of malnutrition. During the FGDs with caregivers of children, it emerged that tube-wells were the main sources of water. However, caregivers consumed water without any purification. Of concern also was that some households had no latrines and even households with toilets did not have sealed latrines. Hand washing practice was also very poor with very few reporting hand washing with soap after defecation or cleaning a child. Though there was not enough evidence of an association with household water source and purification of water with acute malnutrition, the children were less likely to be malnourished if the household had a sanitary latrine (OR 0.34, 95% CI 0.12, 0.95; *p* = 0.039).

The findings of the multivariate conditional logistics regression analysis suggest that birth order, number of family members, illness in the last 2 weeks and household toilet facility are associated with nutritional status of the children (Table 3). A child from large family or who suffered from illness in the last 2 weeks is more likely to be malnourished whereas a child being the first child or having access to sanitary latrine is less likely to be malnourished (Table 3).

**Table 3:** Findings of the multivariate conditional logistic regression exploring association between risk factors and nutritional status of the child, Kurigram, Bangladesh, Feb–May 2014

Covariates	Odds ratio^a^	95% confidence interval	*p* value
Mother’s age (in years)	1.05	0.98, 1.12	0.187
Number of family members	1.30	1.02, 1.65	0.033
Birth Order			
Not first child	1	-	-
First child	0.30	0.09, 0.96	0.042
Illness in the last 2 weeks			
No	1	-	-
Yes	3.08	1.13, 8.42	0.028
Household toilet facility			
Non hygienic latrine	1	-	-
Hygienic latrine	0.25	0.07, 0.82	0.022
Household food security^b^			
Secure	1	-	-
Insecure	2.45	0.82, 7.25	0.106

^a^Odds ratio indicates odds of being acutely malnourished adjusted for other factors in the model taking age group and sex matching into consideration

^b^Household food security was assessed by household food insecurity access scale

## Discussion

The results of the multivariate logistic regression indicate that illness in the last 2 weeks and number of family members are the risk factors of acute malnutrition while being the first child and household access to a hygienic latrine prevents acute malnutrition for children enrolled in a community-based programme in Kurigram. Our qualitative study also revealed that inappropriate feeding practice is perceived as one of the major causes of acute malnutrition by the caregivers of the children.

Black et al. in the Lancet 2008 mentioned diseases as one of the immediate causes of acute malnutrition [1]. The relationship between acute malnutrition and diseases is explained by Ayana et al. as a decrease in appetite and a decrease in the absorption of nutrients from the intestine during illness, which can lead to weight loss [17]. The poor health seeking behaviour of the caregivers’ also contributed a great deal in the process. Similar to our finding, studies in Ethopia, India, and Bangladesh also found significant associations of acute malnutrition with illness, specially diarrhoea and respiratory illness [7, 17, 18].

Considering the numerous health benefits associated with hygiene, Black et al. identified an unhealthy household environment as an underlying cause of acute malnutrition [1]. The relationship with hygienic latrine and control of waterborne diseases, such as diarrhoea, is well stablished. In an evaluation of the Integrated Health–WASH intervention in Kurigram, the authors also reported significant decrease in under 2 child malnutrition over time in the intervention area with better coverage of hygienic latrine compared with only health intervention area [8]. A study conducted in Ethiopia also found association between malnutrition and latrine access [17].

Family size has been documented as a risk factor of malnutrition in several studies in Ethiopia, Pakistan, India, and Malaysia [17–21]. The possible explanation could be children receiving less attention when there are more than one of them.

From 6 months up to 2 years of age and beyond, along with breastfeeding, infants should receive nutritionally adequate and safe complementary foods to meet their evolving and increasing nutritional requirements [22]. However, the caregivers’ lack of awareness, and financial problems could be linked to sub-optimal frequency of complementary feeding which affects the nutrition status of the children. Malnutrition was also associated with food insecurity and food taboos—albeit weak—this association was in line with caregivers’ perspective also. Studies conducted in Bangladesh, India, and Africa also reported association between child malnutrition and inappropriate feeding practice [23–25].

The study was conducted as part of a community-based programme which could be considered a strength as only few studies were conducted to explore risk factors of acute malnutrition based on children enrolled in a community programme. The mixed-method approach to evaluate the association between acute malnutrition to other exposures quantitatively and understand community perspective and practice which influence child nutritional status qualitatively could also be considered a strength. The study relied on children enrolled in the community programme and matched controls based on the information provided by the community workers and caregivers could be argued as a limitation. The small sample size of the study is another limitation in generalizing the findings. Although caregivers from both Tdh intervention and non-intervention areas were recruited for the qualitative part of the study, Tdh’s behaviour change activities may have influenced the community perception. A longitudinal study following children overtime would have been a better study design in establishing the casual path. However, with limited resources and as part of the monitoring and evaluation activities a case-control study was considered most suitable. In order to minimize biases, experienced staff of Tdh’s Monitoring and Evaluation team were trained and assigned for data collection and anthropometric measurement.

The risk factors identified for acute malnutrition in Kurigram can be useful for designing community-based preventive activities. Community awareness activities can be strengthened to improve awareness on appropriate infant and young child feeding practices and benefits of a small family. Interventions to increase coverage and access to hygienic latrines need to be considered by the local government and the non-governmental organisations. Increasing care seeking from the Community Clinic will be useful—this will need a two-pronged intervention—improving the quality of services in the clinic and ensuring that caregivers seek care early for the sick child. The causal framework of acute malnutrition is complex and needs multi-sectoral preventive interventions to improve nutrition status of children.

## Data Availability

The datasets analysed during the current study are available from the corresponding author upon request.

## Abbreviations

ACF: Action Contre la faim
BDHS: Bangladesh Demographic and Health Survey
CHC: Community health clinic
CMAM: Community management of acute malnutrition
FCS: Food consumption score
HFIAS: Household food insecurity access scale
HSS: Household hunger scale
IYCF: Infant and young child feeding
MUAC: Mid upper arm circumference
Tdh: Terre de hommes
WASH: Water sanitation and hygiene
WHO: World Health Organization
WHZ: Weight-for-height *z*-score
